# The long-term trend of uterine fibroid burden in China from 1990 to 2019: A Joinpoint and Age–Period–Cohort study

**DOI:** 10.3389/fphys.2023.1197658

**Published:** 2023-06-22

**Authors:** Xingyu Liu, Bo Wang, Qianyu Zhang, Jinjin Zhang, Shixuan Wang

**Affiliations:** ^1^ Department of Obstetrics and Gynecology, Tongji Hospital, Tongji Medical College, Huazhong University of Science and Technology, Wuhan, China; ^2^ National Clinical Research Center for Obstetrical and Gynecological Diseases, Huazhong University of Science and Technology, Wuhan, China; ^3^ Key Laboratory of Cancer Invasion and Metastasis, Ministry of Education, Huazhong University of Science and Technology, Wuhan, China

**Keywords:** uterine fibroids, mortality, disability-adjusted life years, Joinpoint regression analysis, Age–Period–Cohort analysis

## Abstract

**Purpose:** Uterine fibroids occur in 40%–60% of women and are symptomatic in 30% of the patients by causing abnormal uterine bleeding, pelvic pressure, pain, and infertility. The study aims to evaluate the long-term trend of uterine fibroids mortality and disability-adjusted life years (DALYs) in China and the relative risks of age, period, and birth cohort effects.

**Methods:** The mortality and DALYs of uterine fibroids from 1990 to 2019 were derived from the Global Burden of Disease 2019. The annual percentage change and average annual percent change (AAPC) were assessed using the Joinpoint regression. The effects of age, period, and birth cohort on death and DALYs were analyzed by the Age–Period–Cohort framework.

**Results:** The age-standardized rates were all on the ascending trend, with the greatest increase in the age-standardized mortality rate (AAPC, 1.53; 95% CI, 1.04–2.02). The net drift was found to be 3.51% (95% CI, 2.25%–4.78%) per year for mortality and 0.34% (95% CI, 0.14%–0.53%) per year for DALYs. Significant age, period, and birth cohort effects were found for mortality and DALYs (*p* < 0.001 for all). The mortality risk increased overall with age, but the DALYs risk increased first and then decreased with age. The period and birth cohort risks for mortality and DALYs showed different trends.

**Conclusion:** These secular time trends and changes of mortality and DALYs reveal the socioeconomic alterations, reform of diagnosis and therapy, and changes in social lifestyles and behaviors. Uterine fibroids are still the most common benign gynecological tumors in women, and more epidemiological investigations and social health prevention and control should be applied.

## Introduction

Uterine fibroids are the most common benign gynecological tumors in women (Bulun, 2013; Drayer and Catherino, 2015), which are derived from uterine smooth muscle cells and consist of massive extracellular matrices (Kim and Sefton, 2012). Due to their hormonally responsive nature, fibroids increase with age during a women’s reproductive years, are rare before puberty, and regress after menopause (Wise and Laughlin-Tommaso, 2016). The incidence of uterine fibroids was 60% and 40% by the age of 35 years in African–American women and white women, respectively, growing to >80% and 70% by the age of 50 years (Baird et al., 2003). In 2014, the estimated incidence of uterine fibroids among women was 101.4/10,000 person-years, with the highest incidence rate in the age group of 45–49 years (240.3/10,000 person-year) in the United States (Yu et al., 2018). Besides, the prevalence of fibroids varies from 4.5% to 68.8% depending on different countries and studies (Stewart et al., 2017). Although the data varies widely, uterine fibroids are recognized to affect a large number of women.

Although benign, the lesions of fibroids may lead to the dysfunction of the uterus and cause increased menstrual flow, prolonged periods, anemia, pelvic discomfort, obstruction of fertilized eggs, miscarriage, abnormal fetal positioning, placental abruption, postpartum hemorrhage, preterm labor, and malignancy (Bulun, 2013). Up to 25% of all women and 30%–40% of perimenopausal women may be influenced by symptomatic uterine fibroids (Cramer and Patel, 1990); surgery is the main option for women with severe symptoms. Given the high incidence of uterine fibroids, they are a heavy healthcare burden for women. Nearly 29% of gynecological hospitalizations are attributable to fibroids (Whiteman et al., 2010). In the United States, approximately 200,000 hysterectomies, 30,000 myomectomies, and thousands of uterine artery embolizations and high-intensity focused ultrasound procedures are performed each year for the removal or destruction of fibroids (Bulun, 2013), which occupy 40%–60% of all performed hysterectomies and account for 30% of hysterectomies among women aged 18–44 years (Merrill, 2008).

The high prevalence, hospitalization rates, and surgery rates of uterine fibroids have a profoundly negative impact on healthcare and economic costs worldwide, which include direct costs and indirect costs. The direct annual costs of uterine fibroids, such as the cost of surgeries, hospital admissions, outpatient visits, and medications, are 4.1–9.4 billion dollars (Cardozo et al., 2012). Nevertheless, the indirect annual costs include $1.55–$17.2 billion of the estimated lost work costs and $238 million–$7.76 billion of obstetric outcomes attributed to uterine fibroids (Cardozo et al., 2012). Totally, the costs of uterine fibroids are estimated to be $5.9–$34.4 billion annually in the United States. The mean total cost of hysterectomy for uterine fibroids ranges from 7,370 yuan to 10,064 yuan in China (Gu et al., 2015). Obviously, uterine fibroids remain a problematic issue for women’s healthcare and health economies, especially in a developing country like China with a large number of women. However, relevant studies have scarcely evaluated the long-term trend of uterine fibroids in China. Moreover, the potential cause of the long-term trend is underdetermined, and the relative risks of age, period, and birth cohort effects remain to be elucidated.

To gain insight into this area, we aim to interrogate the long-term trend in death and disability-adjusted life years (DALYs) and investigate the specific effects of age, period, and birth cohort in China from 1990 to 2019 using the Joinpoint regression and Age–Period–Cohort analyses, based on detailed and comparable information of the Global Burden of Disease 2019 (GBD 2019) (Mattiuzzi and Lippi, 2020). Our findings may provide evidence for uterine fibroids intervention by integration and allocation of health resources.

## Materials and methods

### Data resource

The mortality and DALYs of uterine fibroids from 1990 to 2019 were derived from the GBD 2019, which provides a comprehensive and internally consistent estimation of the health losses from hundreds of diseases, injuries, and risk factors to improve health systems and eliminate disparities (Lancet, 2018a; Lancet, 2018b). The GBD 2019 comprised assessments of age–sex-specific all-cause and cause-specific mortality and DALYs from 369 diseases and 87 risk factors in 204 countries and territories (Lancet, 2020a; Lancet, 2020b). The methods of GBD 2019 were fully understood as reported previously (GBD, 2019 Demographics Collaborators, 2020; Lancet, 2020a). Uterine fibroids were diagnosed based on the International Statistical Classification of Diseases and the International Classification of Diseases and Injuries (ICD-10). The mortality and DALY rates of uterine fibroids in Chinese women were age-standardized based on the GBD global age-standardized population (Zhou et al., 2016). The study enrolled a national population–based Chinese cohort from the GBD, and no interaction with human subjects or personal private information was involved.

### Statistical analysis

#### Joinpoint regression analysis

Trends of the mortality or DALY rate over a specific time interval could be depicted by a Joinpoint regression model under the assumption that the change of rates is constant over each time partition defined by the transition points, but varies among different time partitions (Clegg et al., 2009). The basic theory of the Joinpoint regression model is to separate the whole specific-time-interval regression trend into several segmented regression trends by transition points, of which the maximum number is five. This analysis postulates that dependent variables follow a Poisson distribution, exploiting the year as the predictor (Iannuzzi et al., 2022). The significance level of each segmented regression trend was evaluated by using Monte Carlo methods and the significance level of the whole specific-time-interval regression trend was obtained by a Bonferroni correction (Clegg et al., 2009). The segmented regression trends were presented as the annual percentage change (APC) and 95% confidence intervals (CI), while the whole specific-time-interval regression trend was presented as the average annual percent change (AAPC) and 95% CI. The Joinpoint Regression Program 4.9.1.0 (National Cancer Institute, Washington, DC) was used to calculate the APC and AAPC. A two-tailed *p-*value < 0.05 was considered statistically significant.

#### Age–Period–Cohort analysis

The Age–Period–Cohort (APC) model was designed to evaluate the effects of age, period, and birth cohort on outcomes, especially in the sociology and epidemiology fields. It is based on a log-linear model for the outcome variable with additional effects from the age, period, and birth cohort, as shown in the following formula:

$$Y_{ijk}=u+\alpha_{i}+\beta_{j}+\gamma_{k}{+\varepsilon }_{ijk}.$$

where $Y_{ijk}$ is the outcome variable, such as the mortality rate; μ is the intercept; $\alpha_{i}$ represents the *i*-th age effect; $\beta_{j}$ represents the *j*-th period effect; $\gamma_{k}$ represents the *k*-th birth cohort effect; and $\varepsilon_{ijk}$ is the error term (Fosse and Winship, 2019). In this model, the age effect represents the effect of outcome risk changes related to different age groups; the period effect represents the effect of outcome risk changes of all age groups at different time intervals; the birth cohort effect represents the effect of outcome risk changes associated with different birth cohorts (Yang and Land, 2013). Net drift, the overall logarithmic linear trend by period and birth cohort, indicates the overall annual percentage change; local drifts, the logarithmic linear trend by period and birth cohort for each age group, indicate annual percentage changes for each age group; longitudinal age curve indicates the fitted longitudinal age-specific rates in the reference birth cohort adjusted for period deviations; the period relative risk (RR) indicates the RR adjusted for age and non-linear birth cohort effects in a certain period versus the reference period, which represents the period effect; the birth cohort RR indicates the relative risk adjusted for age and non-linear period effects in a certain birth cohort versus the reference birth cohort, which represents the birth cohort effect (Rosenberg et al., 2014; Wang et al., 2017; Wang et al., 2021).

For the APC analysis, the mortality and population data for uterine fibroids were divided into continuous 5-year periods from 1990 to 2019 and consecutive 5-year age intervals from 10–14 to 90–94 years. Uterine fibroids were rare in age groups under 10 years, and the data for age groups over 94 years in China were not provided in GBD 2019. The age-specific rates were appropriately recoded into successive 5-year age groups (10–14, 15–19, …, and 90–94 years), consecutive 5-year periods from 1990 to 2019 (1990–1994, 1995–1999, …, and 2015–2019), and correspondingly consecutive 5-year birth cohorts (1898–1902, 1903–1907, …, 1998–2002, and 2003–2007) to estimate net age, period, and birth cohort effects of uterine fibroids mortality and DALYs. The central period (2000–2004) and birth cohort (1948–1952) were defined as the references, respectively. The estimable parameters were obtained by using the APC web tool (Biostatistics Branch, National Cancer Institute, Bethesda, MD, http://analysistools.nci.nih.gov/apc/) (Rosenberg et al., 2014). The central age group, period, and birth cohort were defined as the respective references in the APC analysis. The significance of the estimable functions was tested by the Wald chi-squared test. Other analyses were performed by the R Project for Statistical Computing (version 4.0.5, http://www.r-project.org/). All statistical tests were two-tailed, and a *p-*value < 0.05 was considered statistically significant.

## Results

### Trends of uterine fibroids incidence, death, and DALY rates in China

**FIGURE 1:**
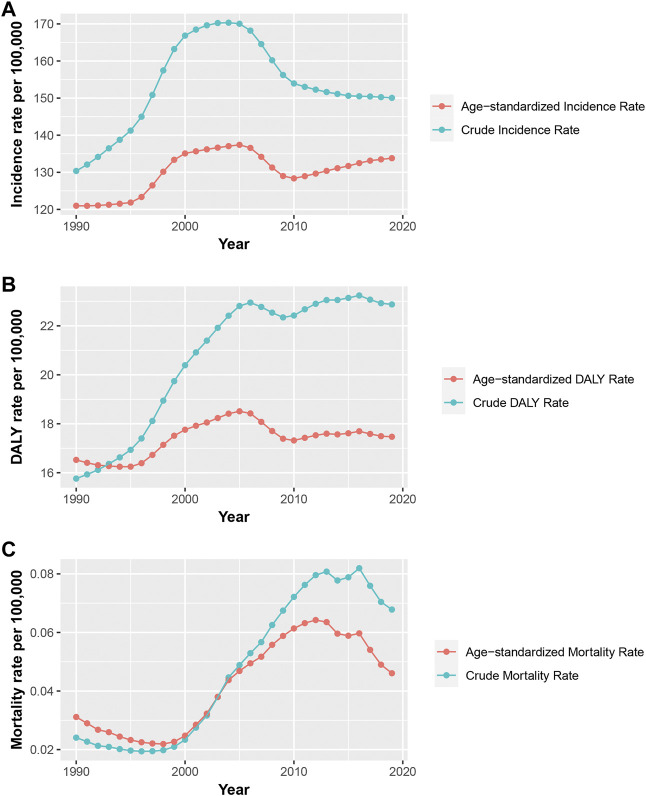
Trends of the crude rates and the age-standardized rates per 100,000 persons for incidence **(A)**, mortality **(B)**, and DALY **(C)** of uterine fibroids in China, 1990–2019 **(A)**, The crude rates and the age-standardized rates per 100,000 persons for incidence of uterine fibroids in China, 1990–2019; **(B)**, The crude rates and the age-standardized rates per 100,000 persons for mortality of uterine fibroids in China, 1990–2019; **(C)**, The crude rates and the age-standardized rates per 100,000 persons for DALY of uterine fibroids in China, 1990–2019; DALY, disability-adjusted life years.

By and large, there were 747,648 incidences, 138 deaths, and 90,389 DALYs in China in 1990 and 1,046,738 incidences, 473 deaths, and 159,558 DALYs in China in 2019, with a 40.0% increase in incidence, 242.8% increase in death, and 75.6% increase in DALYs. Trends of uterine fibroids incidence, death, and DALY rates in China from 1990 to 2019 are shown in Figure 1. In general, the crude incidence rate and age-standardized incidence rate (ASIR) exhibited a binormal distribution with peaks in 2004 and 2005. The crude mortality rate and age-standardized mortality rate (ASMR) were elevated fast from 2000 and began to fall since 2013. The crude DALY rate and age-standardized mortality rate showed an obvious upward trend since 1990 and stabilized after 2000.

Table 1 displays the AAPCs and APCs in uterine fibroids incidence, death, and DALYs in China from 1990 to 2019. The age-standardized rates were all on the ascending trend, with the greatest increase in ASMR (AAPC, 1.53; 95% CI, 1.04–2.02). As shown in Supplementary Table S1, for age-specific incidence rates, significant increases were observed in the 30–34, 35–39, and 40–44 years age groups, in which age-specific DALY rates also showed significant increases. A stable trend was observed in the 25–29 years age group, while the other age groups were on the decline. The age-specific mortality rates were on the ascending trend in most age groups. The age-specific DALY rates were on the decline in the younger age groups (10–14, 15–19, 20–24, and 25–29 years) and on the increase in the older age groups (75–79,80–84, 85–89, and 90–94 years).

**TABLE 1:** Trends of ASIR, ASMR, and age-standardized DALY rates in uterine fibroids in China.

ASR	Period	APC (95% CI)	*p*-value
ASIR			
Trend1	1990–2004	1.17 (0.99, 1.35)	<0.001
Trend2	2004–2010	−1.30 (−2.11, −0.50)	0.003
Trend3	2010–2019	0.50 (0.19, 0.82)	0.003
AAPC	1990–2019	0.45 (0.24, 0.65)	<0.001
ASMR			
Trend1	1990–1999	−3.66 (−4.60, −2.71)	<0.001
Trend2	1999–2004	16.00 (13.85, 18.20)	<0.001
Trend3	2004–2011	5.63 (5.11, 6.16)	<0.001
Trend4	2011–2016	−2.02 (−3.00, −1.04)	0.001
Trend5	2016–2019	−7.92 (−9.44, −6.38)	<0.001
AAPC	1990–2019	1.53 (1.04, 2.02)	<0.001
Age-standardized DALY rate			
Trend1	1990–1995	−0.39 (−0.54, −0.24)	<0.001
Trend2	1995–2000	1.90 (1.68, 2.12)	<0.001
Trend3	2000–2005	0.94 (0.72, 1.16)	<0.001
Trend4	2005–2009	−1.69 (−2.01, −1.37)	<0.001
Trend5	2009–2016	0.23 (0.12, 0.34)	<0.001
Trend6	2016–2019	−0.41 (−0.74, −0.09)	0.016
AAPC	1990–2019	0.20 (0.12, 0.27)	<0.001

Abbreviations: ASR, age-standardized rate; ASIR, age-standardized incidence rate; ASMR, age-standardized mortality rate; APC, annual percentage change; AAPC, average annual percent change; CI, confidence interval; DALYs, disability-adjusted life years.

### Effect of age, period, and birth cohort on uterine fibroid mortality and DALY

**FIGURE 2:**
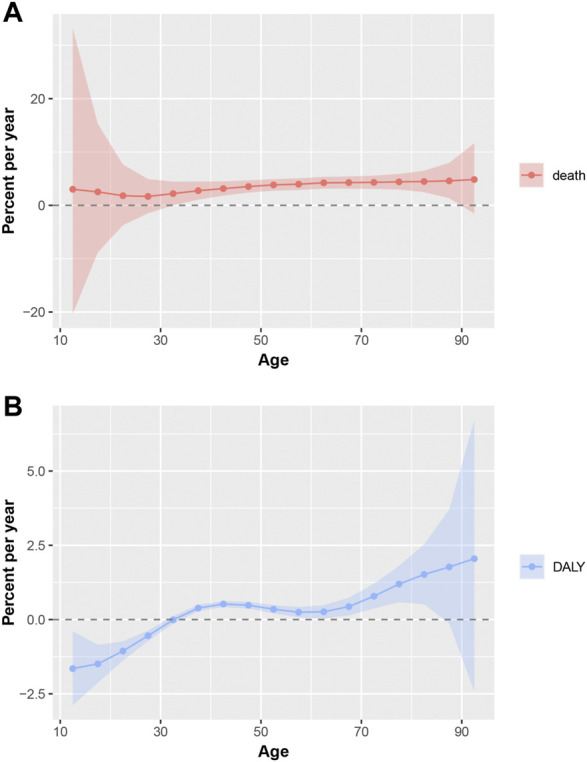
Local drifts of mortality and DALY for uterine fibroids in China. Age group-specific annual percent change (%) in mortality and DALY rate and the corresponding 95% CI. **(A)**, Local drifts of mortality for uterine fibroids in China; **(B)**, Local drifts of DALY for uterine fibroids in China; DALY, disability-adjusted life years.

The net drift indicating the overall annual percentage change was 3.51% (95% CI, 2.25%–4.78%) per year for mortality and 0.34% (95% CI, 0.14%–0.53%) per year for DALY. The local drifts, which represent annual percentage changes for each age group, are shown in Figure 2. The local shift values of mortality were above 0 in all age groups (Figure 2A). The local shift value of DALY exhibited an overall upward trend, where the values were below 0 for younger age groups (10–14, 15–19, 20–24, 25–29, and 30–34 years) and above 0 for age groups over 34 years (Figure 2B).

**FIGURE 3:**
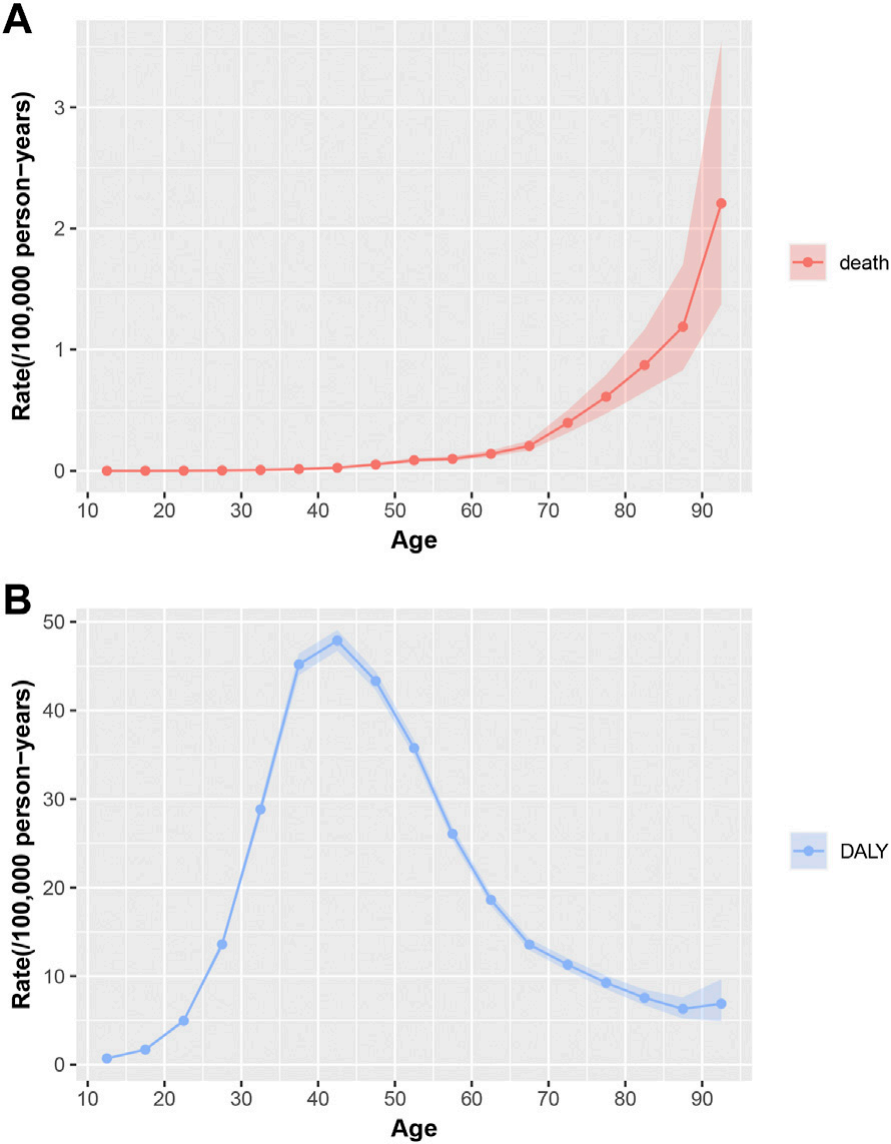
Longitudinal age curves of mortality and DALY for uterine fibroids in China. Fitted longitudinal age-specific rates of mortality and DALY (per 100,000 person-years) and the corresponding 95% CI. **(A)**, Longitudinal age curves of mortality for uterine fibroids in China; **(B)**, Longitudinal age curves of DALY for uterine fibroids in China; DALY, disability-adjusted life years; CI, confidence interval.

Figure 3 displays the longitudinal age curves of uterine fibroids mortality and DALY. Chinese women in the same birth cohort witnessed a stable growth in the mortality risk for uterine fibroids with age (Figure 3A). Contrary to the steady growth of mortality, DALYs showed a binormal distribution with a peak in the 40–44 years age group (Figure 3B).

**FIGURE 4:**
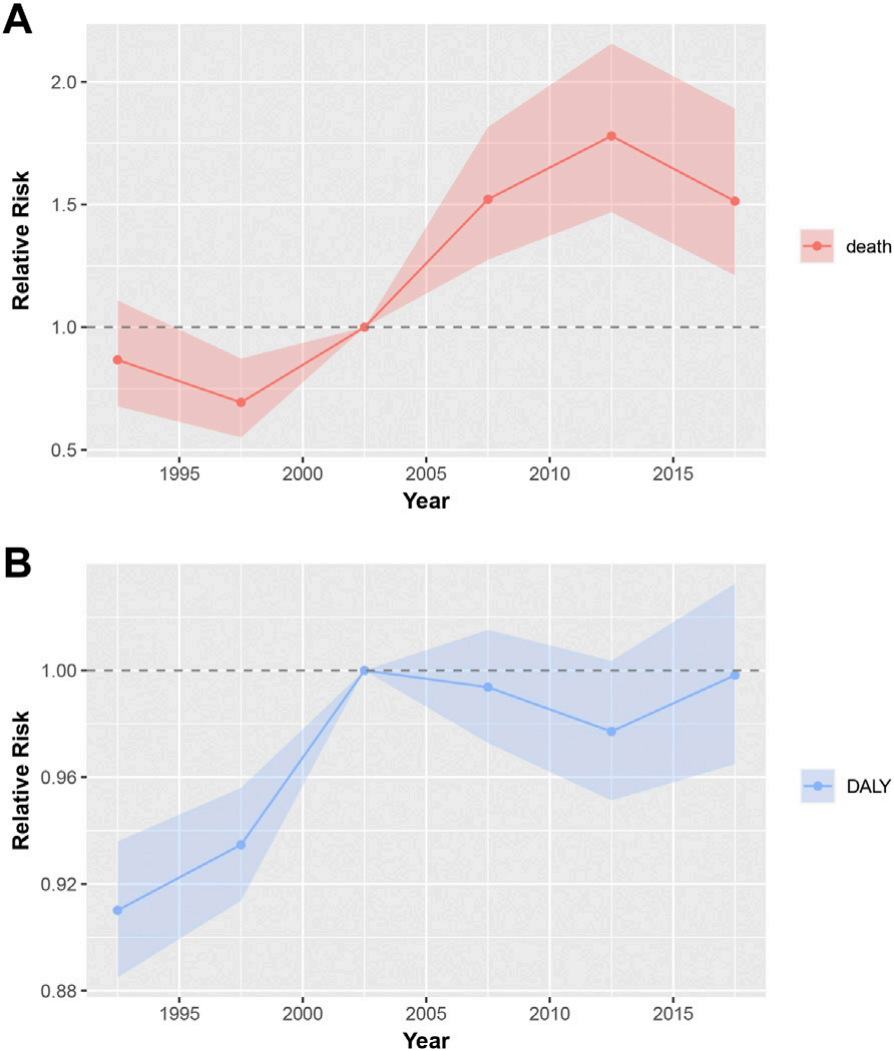
Period RRs of mortality and DALY for uterine fibroids in China. The RR of each period adjusted for age and non-linear cohort effects and the corresponding 95% CI. **(A)**, Period RRs of mortality for uterine fibroids in China; **(B)**, Period RRs of DALY for uterine fibroids in China; DALY, disability-adjusted life years; RR, relatively risk; CI, confidence interval.

The estimation of the period RR of mortality and DALYs is shown in Figure 4. The mortality of uterine fibroids shows an increasing trend between the period 1995 and 2015 and a declining trend for the periods 1990–1994 and 2015–2019 (Figure 4A). The mortality RR increased by approximately 74.6% in the period 2015–2019 when compared to that in the period 1990–1994. The DALY RRs of uterine fibroids were on an increase for most periods except for the period 2005–2014 (Figure 4B). When compared to the reference period 2000–2004, the period RRs of DALYs in other periods did not differ much. Detailed information on the period RR is shown in Supplementary Table S2.

**FIGURE 5:**
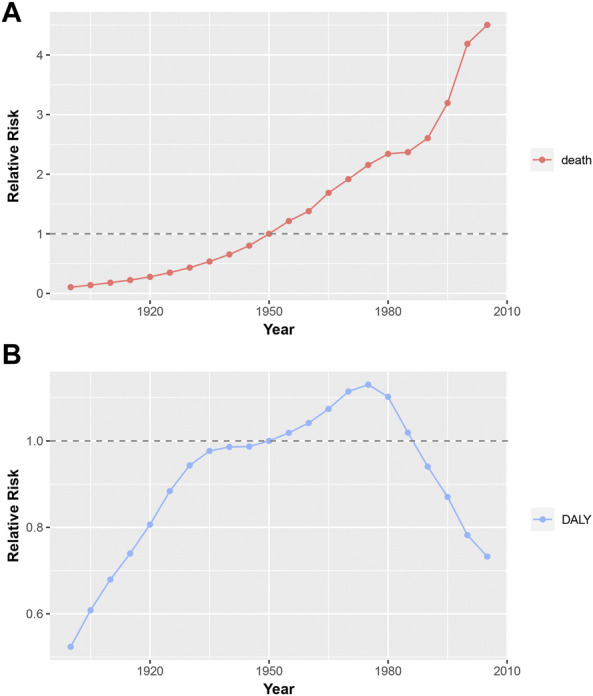
Birth cohort RRs of mortality and DALY for uterine fibroids in China. The RR of each birth cohort adjusted for age and non-linear period effects. **(A)**, Birth cohort RRs of mortality for uterine fibroids in China; **(B)**, Birth cohort RRs of DALY for uterine fibroids in China; DALY, disability-adjusted life years; RR, relatively risk.

The estimated birth cohort RR of mortality and DALYs is displayed in Figure 5. The overall trend of mortality RR is on the increase (Figure 5A). Compared with the birth cohort 1898–1902, the RR of mortality in the birth cohort 2003–2007 is increased by 427.2% for uterine fibroids. Different from the steady growth of mortality, the DALY risk shows a binormal distribution with a peak in the birth cohort 1973–1977 (Figure 5B). The RRs of DALYs in the birth cohort 1973–1977 is increased by 115.8% when compared to that in the birth cohort 1898–1902. Detailed information on the period RR is shown in Supplementary Table S2.

Finally, the net drifts of mortality and DALYs and the local shifts of DALYs are statistically significant according to the Wald test results (*p-*value < 0.001 for all), and the birth cohort and period RRs of mortality and DALYs were also statistically significant (*p-*value < 0.001 for all). However, the local shifts of death are not significant (*p* = 0.999).

## Discussion

To our knowledge, this is the first study to investigate the long-term trend of mortality and DALYs of uterine fibroids in China from 1990 to 2019 and evaluate the specific effects of age, period, and birth cohort based on the APC framework analysis. The trends of incidence, mortality, and DALY rates in China were not consistent with the trends of the corresponding global rates (Cheng et al., 2022). When compared with an overall upward trend in ASIR and downward trends in ASMR and age-standardized DALY rate at the global level, the ASIR, ASMR, and age-standardized DALY rates of uterine fibroids in China fluctuated, increased, and decreased, respectively, over the past 30 years. Overall, the age-standardized rates were all on the ascending trend, with the greatest increase in ASMR (AAPC, 1.53; 95% CI, 1.04–2.02). The findings of our study provide valuable evidence for uterine fibroids control through policy and health system planning.

Uterine fibroids are the most common and frequently occurring benign gynecological tumors in women of productive age. Increasing age is one of the most important risk factors for uterine fibroids, especially in women aged over 40 years and women at the premenopausal stage (Faerstein et al., 2001; Wise et al., 2005). According to the longitudinal age curve results of mortality and DALYs, it is clear that the mortality risk increased overall with age, but the DALY risk increased first and then decreased with age. Since DALY is mainly influenced by incidence, the incidence of uterine fibroids reaches a peak at premenopausal age. The peak of the DALY risk was in the 40–44 years age group.

The period effects reflected the influence of different time periods on death and DALYs among all age groups, generally through social, economic, and medical measures, especially the changes in guidelines. So, when we wonder about the reasons for the mortality risk due to period effects, the impact of the International Classification of Diseases (ICD) coding issues should be taken into consideration (Wang et al., 2017). The improvement in cause-of-death reporting in China may be closely associated with the increase in the period RRs of mortality for uterine fibroids. Uterine fibroids–related deaths were possibly underestimated when the quality of reporting of the cause-of-death codes was suboptimal in the past. For instance, an old woman with uterine fibroids had likely died of severe anemia, massive uterine bleeding, or other complications; however, the physician responsible for reporting the cause-of-death may have ignored fibroids and instead filled in the underlying cause of death as severe anemia, massive uterine bleeding, or other fatal illness. A similar phenomenon was found in a study of reported causes of injury and poisoning deaths in China (Gao and Li, 2013). The decline of DALY RRs after 2004 might be due to improvements of therapy; advancements in medical and uterine-preserving treatment options have been made in the past decade. A step-up approach, beginning with pharmacological and minimally invasive treatments before moving to surgery, is recommended by many international obstetrical and gynecology societies when treating uterine fibroids (ACOG practice bulletin, 2008). Available medical therapies include non-hormonal treatment and hormonal treatment, which work together to manage symptoms. Minimally invasive treatments such as uterine artery embolization and magnetic resonance–guided focused radiofrequency ablation are shorter procedures with faster recovery and quicker return to normal activities (ACOG practice bulletin, 2008). These improvements in medical and minimally invasive therapy have helped decrease the DALYs in recent years.

The birth cohort effects reflect different risk factors in early life, such as environmental, behavioral, and socioeconomic factors. The birth cohort RRs of mortality increased gradually in all birth cohorts, and the birth cohort RRs of DALY increased before the birth cohort 1973–1977 and then decreased somehow. The increase in mortality might be mainly due to unhealthy lifestyles. Obesity prevalence and the rate of increase have been high in women (Afshin et al., 2017). The rapid growth of obesity in women probably leads to an increase in the occurrence and growth of uterine fibroids through hormonal and inflammatory mechanisms (Wise and Laughlin-Tommaso, 2016; Ciavattini et al., 2017). Apart from obesity, the alteration of lifestyle might also increase the risk of uterine fibroids. Less physical activity, stress of work and family, unhealthy diet, smoking, alcohol, and too much caffeine consumption all contribute to a higher risk of uterine fibroids (Pavone et al., 2018). Since fibroids are estrogen related, birth control methods (such as oral contraceptives and intrauterine devices) for the policy of one child per family might be partially responsible for the birth cohort risk increase in DALYs before the birth cohort 1973–1977 (Wang, 2012). The possible reason for the following decline in DALY period risks might be the widespread use of condoms.

Strengths of this study include a comprehensive and up-to-date analysis of uterine fibroid death and DALYs in China between 1990 and 2019 and the illustrated effects of age, period, and birth cohort through the Age–Period–Cohort analysis. Due to the inherent deficiencies of GBD 2019, some limitations are unavoidable in this study. First, the accuracy of the results of this study depends on the quality and quantity of GBD data, although there were many adjustment steps, such as the correction of incompleteness, underreporting, and misclassification, as well as redistribution of garbage codes, to improve data quality and comparability of data in GBD 2019. Second, due to the limitation of GBD 2019 data, we could not investigate the incidence and burden of uterine fibroids subtypes by location (intermural, subplasma, and submucosa). Third, since the GBD study took the country as its basic unit, we cannot investigate the differences in race. Finally, GBD 2019 only includes DALY, YLL, and YLD as the indicators of disease burden, therefore the recurrence rate, malignancy rate, and impact on fertility cannot be estimated.

The study investigates the secular time trends of mortality and the DALY trend of uterine fibroids from 1990 to 2019 in China. The age-standardized rates were all on the ascending trend, with the greatest increase in ASMR. The net drift was found to be 3.51% (95% CI, 2.25%–4.78%) per year for mortality and 0.34% (95% CI, 0.14%–0.53%) per year for DALYs. Significant age, period, and birth cohort effects were found for mortality and DALYs. These secular time trends and effects of mortality and DALYs reveal socioeconomic alterations, reforms of diagnosis and therapy, and changes in social lifestyles and behaviors. More epidemiological investigations and social health prevention and control should be applied to uterine fibroids. Our results suggest an exaggeration of the epidemic among older individuals. Timely intervention should be conducted, especially for earlier birth cohorts at high risk.

## Data Availability

The data sets presented in this study can be found in online repositories. The names of the repository/repositories and accession number(s) can be found at http://ghdx.healthdata.org/gbd-results-tool.
