# Supplementary material for: The long-term trend of uterine fibroid burden in China from 1990 to 2019: A Joinpoint and Age–Period–Cohort study
Source: Front Physiol. 2023 Jun 22;14:1197658. doi: 10.3389/fphys.2023.1197658 (PMC10324033; doi:10.3389/fphys.2023.1197658)
Supplement: Supplementary file 1 [file Table1.DOCX]

**Supplementary files**

**Supplementary Table S1. The average annual percent changes (AAPCs) of death and DALY from 1990 to 2019 for uterine fibroids in China.**

**Supplementary Table S2. The mortality and DALY relative risks of uterine fibroids in China due to period and birth cohort effects.**

**Supplementary Table S1. The average annual percent changes (AAPCs) of death and DALY from 1990 to 2019 for uterine fibroids in China.**

| **Age Group** | **Incidence**  **(AAPC,95%CI)** | ***P* value** | **Death**  **(AAPC,95%CI)** | ***P* value** | **DALY**  **(AAPC,95%CI)** | ***P* value** |
| --- | --- | --- | --- | --- | --- | --- |
| ASR | 0.45 (0.24, 0.65) | < 0.001 | 1.53 (1.04, 2.02) | < 0.001 | 0.20 (0.12, 0.27) | < 0.001 |
| 10-14 | -1.06 (-1.19, -0.94) | < 0.001 | 1.98 (0.73, 3.25) | 0.002 | -1.04 (-1.15, -0.92) | < 0.001 |
| 15-19 | -0.86 (-0.95, -0.76) | < 0.001 | 1.17 (-0.10, 2.46) | 0.072 | -0.97 (-1.16, -0.78) | < 0.001 |
| 20-24 | -0.28 (-0.41, -0.16) | < 0.001 | 0.12 (-0.75, 1.00) | 0.790 | -0.70 (-0.79, -0.61) | < 0.001 |
| 25-29 | -0.03 (-0.17, 0.11) | 0.716 | 0.15 (-0.66, 0.96) | 0.719 | -0.38 (-0.50, -0.26) | < 0.001 |
| 30-34 | 0.55 (0.51, 0.59) | < 0.001 | 0.85 (0.01, 1.69) | 0.047 | 0.01 (-0.12, 0.13) | 0.908 |
| 35-39 | 0.94 (0.89, 1.00) | < 0.001 | 0.59 (0.10, 1.09) | 0.019 | 0.42 (0.34, 0.50) | < 0.001 |
| 40-44 | 0.55 (0.52, 0.59) | < 0.001 | 1.00 (0.39, 1.61) | 0.001 | 0.48 (0.36, 0.60) | < 0.001 |
| 45-49 | -0.76 (-0.85, -0.68) | < 0.001 | 1.52 (0.75, 2.31) | < 0.001 | 0.41 (0.24, 0.58) | < 0.001 |
| 50-54 | -1.43 (-1.58, -1.28) | < 0.001 | 1.04 (0.43, 1.66) | 0.001 | 0.12 (0.03, 0.21) | 0.009 |
| 55-59 | -1.79 (-1.96, -1.61) | < 0.001 | 1.08 (0.28, 1.88) | 0.008 | -0.02 (-0.19, 0.15) | 0.811 |
| 60-64 | -2.02 (-2.17, -1.87) | < 0.001 | 1.42 (0.41, 2.44) | 0.006 | -0.02 (-0.19, 0.15) | 0.836 |
| 65-69 | -2.05 (-2.20, -1.89) | < 0.001 | 1.54 (1.00, 2.08) | < 0.001 | -0.04 (-0.25, 0.17) | 0.717 |
| 70-74 | -2.08 (-2.23, -1.93) | < 0.001 | 2.01 (1.50, 2.52) | < 0.001 | 0.25 (-0.06, 0.56) | 0.11 |
| 75-79 | -2.13 (-2.28, -1.97) | < 0.001 | 2.05 (1.42, 2.69) | < 0.001 | 0.40 (0.10, 0.70) | 0.008 |
| 80-84 | -1.97 (-2.12, -1.82) | < 0.001 | 2.07 (1.49, 2.65) | < 0.001 | 0.56 (0.28, 0.83) | < 0.001 |
| 85-89 | -1.71 (-1.82, -1.60) | < 0.001 | 2.19 (1.70, 2.69) | < 0.001 | 0.64 (0.37, 0.90) | < 0.001 |
| 90-94 | -1.57 (-1.71, -1.44) | < 0.001 | 2.49 (1.75, 3.23) | < 0.001 | 1.11 (0.71, 1.53) | < 0.001 |

Abbreviations: ASR, age−standardized rate; AAPC, average annual percent change; CI, confidence interval; DALY, disability-adjusted life years.

**Supplementary Table 2. The mortality and DALY relative risks of uterine fibroids in China due to period and birth cohort effects.**

| **Factor** | **Death (RR,95%CI)** | **DALY (RR,95%CI)** |
| --- | --- | --- |
| **Period** | | |
| 1990-1994 | 0.87 (0.68, 1.11) | 0.91 (0.89, 0.94) |
| 1995-1999 | 0.69 (0.55, 0.87) | 0.93 (0.91, 0.96) |
| 2000-2004 | 1.00 (1.00, 1.00) | 1.00 (1.00, 1.00) |
| 2005-2009 | 1.52 (1.28, 1.82) | 0.99 (0.97, 1.02) |
| 2010-2014 | 1.78 (1.47, 2.16) | 0.98 (0.95, 1.00) |
| 2015-2019 | 1.51 (1.21, 1.89) | 1.00 (0.96, 1.03) |
| **Birth Cohort** | | |
| 1898-1902 | 0.11 (0.01, 0.87) | 0.52 (0.12, 2.37) |
| 1903-1907 | 0.14 (0.05, 0.40) | 0.61 (0.33, 1.13) |
| 1908-1912 | 0.18 (0.10, 0.34) | 0.68 (0.49, 0.93) |
| 1913-1917 | 0.22 (0.14, 0.35) | 0.74 (0.61, 0.89) |
| 1918-1922 | 0.28 (0.19, 0.40) | 0.81 (0.71, 0.91) |
| 1923-1927 | 0.35 (0.26, 0.47) | 0.88 (0.81, 0.97) |
| 1928-1932 | 0.43 (0.33, 0.57) | 0.94 (0.88, 1.01) |
| 1933-1937 | 0.54 (0.42, 0.68) | 0.98 (0.93, 1.03) |
| 1938-1942 | 0.65 (0.52, 0.82) | 0.99 (0.95, 1.03) |
| 1943–1947 | 0.8 (0.65, 0.99) | 0.99 (0.95, 1.02) |
| 1948–1952 | 1.00 (1.00, 1.00) | 1.00 (1.00, 1.00) |
| 1953–1957 | 1.21 (1.00, 1.48) | 1.02 (0.99, 1.05) |
| 1958–1962 | 1.38 (1.10, 1.73) | 1.04 (1.01, 1.07) |
| 1963–1967 | 1.69 (1.33, 2.14) | 1.07 (1.05, 1.10) |
| 1968–1972 | 1.92 (1.44, 2.55) | 1.11 (1.08, 1.14) |
| 1973–1977 | 2.15 (1.49, 3.12) | 1.13 (1.1, 1.16) |
| 1978–1982 | 2.34 (1.43, 3.84) | 1.10 (1.06, 1.14) |
| 1983–1987 | 2.37 (1.22, 4.59) | 1.02 (0.98, 1.06) |
| 1988–1992 | 2.60 (0.99, 6.88) | 0.94 (0.89, 1.00) |
| 1993–1997 | 3.19 (0.54, 18.73) | 0.87 (0.78, 0.97) |
| 1998–2002 | 4.19 (0.09, 188.55) | 0.78 (0.63, 0.97) |
| 2003-2007 | 4.50 (0, 20184.91) | 0.73 (0.48, 1.11) |

Abbreviations: RR, relative risks; CI, confidence interval; DALY, disability-adjusted life years.
